# Increase in bone metabolic markers and circulating osteoblast-lineage cells after orthognathic surgery

**DOI:** 10.1038/s41598-019-56484-x

**Published:** 2019-12-27

**Authors:** Yoko Abe, Mirei Chiba, Sanicha Yaklai, Roan Solis Pechayco, Hikari Suzuki, Tetsu Takahashi

**Affiliations:** 1 Division of Oral Physiology, Department of Oral Function and Morphology, Graduate School of Dentistry, Tohoku University, 4-1 Seiryo-machi, Aoba-ku, Sendai 980-8575 Japan; 2 Division of Oral and Maxillofacial Surgery, Department of Oral Medicine and Surgery, Graduate School of Dentistry, Tohoku University, 4-1 Seiryo-machi, Aoba-ku, Sendai 980-8575 Japan

**Keywords:** Bone, Metabolism

## Abstract

Increased mineralisation rate and bone formation after surgery or fracture is the regional acceleratory phenomenon (RAP), and its systemic impact is the systemic acceleratory phenomenon (SAP). The proportion of circulating osteoblast lineage cells, including osteocalcin-positive (OCN^+^) cells, in the peripheral blood is markedly higher during pubertal growth and in patients with bone fractures. This study aimed to elucidate the dynamic changes in bone metabolic activity after orthognathic surgery by longitudinal prospective observation. Peripheral venous blood samples were collected from patients who had undergone orthognathic surgery, and serum bone metabolic markers and the proportion of OCN^+^ cells were measured. Orthognathic surgery induces systemic dynamic changes in bone metabolic activity by targeting steps in the bone healing process and related proteins, such as surgical stress/inflammation (C-reactive protein), bone resorption (type I collagen C-telopeptide), and bone formation (alkaline phosphatase and bone-specific alkaline phosphatase). During the early post-operative period, the population of OCN^+^ cells significantly increased. Confocal microscopy revealed that OCN proteins were localised in the cytoplasm in Triton X-100-treated OCN^+^ cells. Furthermore, *OCN*, *ALP*, and *COL1A1* gene expression was detected in OCN^+^ cells, suggesting the contribution of the local maturation of bone marrow-derived OCN^+^ cells at the site of bone healing.

## Introduction

Mineralisation rate and bone formation increase after surgery and fracture. After proving this phenomenon, Frost^1^ proposed the regional acceleratory phenomenon (RAP), in which healing of hard and soft tissues is accelerated by noxious stimuli (e.g., surgery, fracture). Furthermore, Mueller *et al*.^2^ and Schilling *et al*.^3^ proposed the systemic acceleratory phenomenon (SAP), showing that bone remodelling increased systemically due to RAP. The systemic reaction occurred in the background of bone repair and regeneration mediated by growth factors and cytokines^4,5^.

Stromal stem cells present in the bone marrow can differentiate into broad-spectrum cells, such as osteogenic and hematopoietic cells^6^. Additionally, a population of osteogenic, nonadherent cells in the bone marrow that was osteocalcin-positive (OCN^+^), bone-specific alkaline phosphatase-positive (BAP^+^), or both was identified^7,8^. Circulating OCN^+^ osteoblast lineage cells significantly increased in human peripheral blood during pubertal growth as well as in 3 adult patients with fracture; these cells were proven to form mineral deposits both *in vitro* and *in vivo*^9^ and are expected to play an important role in the healing process of fractures^5,10^.

Injuries involve inflammation and hypoxia, which induce stromal cell-derived factor-1 (SDF-1)^11,12^ and bone morphogenetic proteins (BMPs)^13,14^. SDF-1 and BMPs form a chemoattractant gradient and recruit circulating osteogenic precursor (COP) cells, such as those expressing CXCR4 from bone marrow, via circulation. Finally homing of COP cells ossifies cartilage^10^.

Bone healing after bone splitting by orthognathic surgery and that after bone splitting by fracture are thought to be similar^15^. However, the detailed mechanism of healing after orthognathic surgery, especially in humans, is unclear. The degree of the effect; whether it is regional or systemic; whether it lasts for several days, weeks, or months; the mechanism by which not only catabolic but also anabolic effects occur; and whether circulating OCN^+^ cells associate after surgery like after fractures are all unclear.

Thus, this study aimed to determine the dynamic changes of serum bone metabolic markers and circulating OCN^+^ cells in human peripheral blood during bone repair after orthognathic surgery. Our data provide evidence directly linked to clinical practice.

## Results

The study included 28 consecutive patients (10 men and 18 women, 18–40 years of age, mean age: 23.0 ± 5.4 years) undergoing orthognathic surgery. No patient had wound infections, non-union, or failures in wound healing after orthognathic surgery during follow-up as determined by X-ray findings and clinical examination. None had additional surgical treatment, tooth extraction, or bone metabolic diseases nor received medications affecting bone metabolism.

### Changes in serum metabolic markers after orthognathic surgery

**Figure 1:**
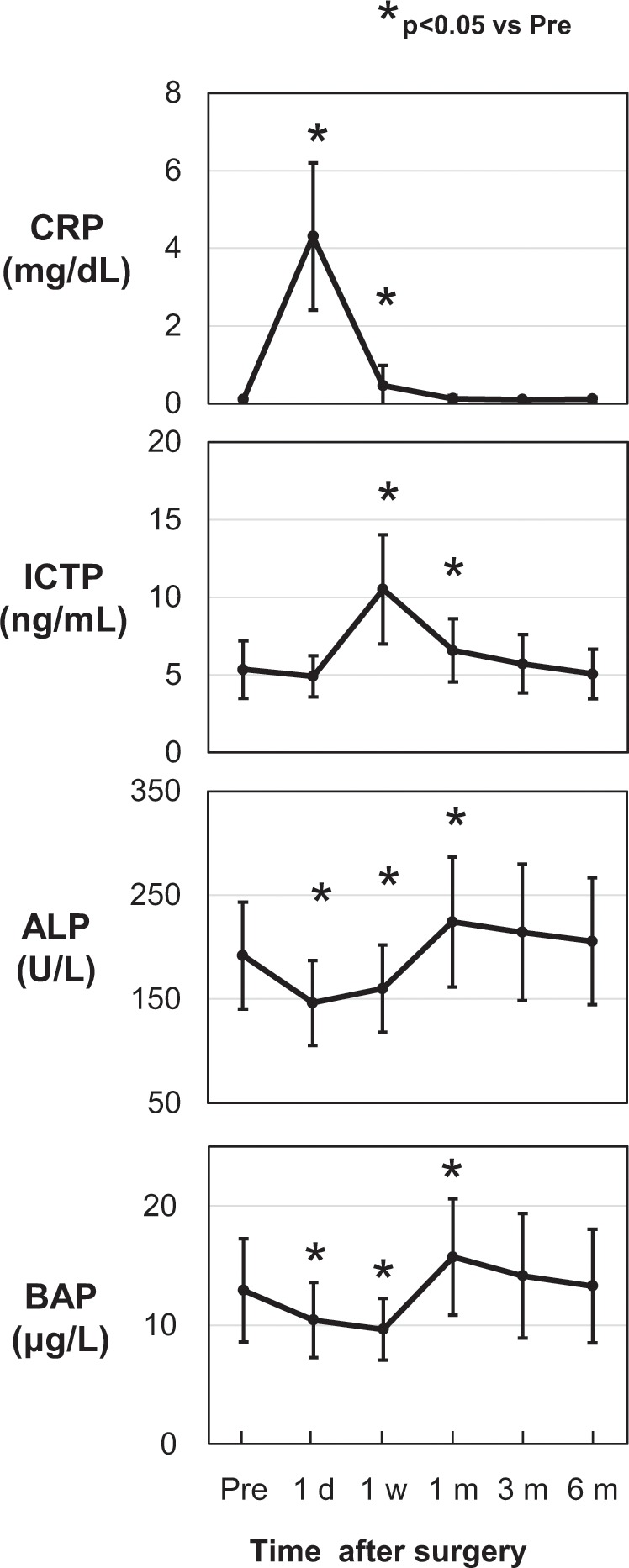
Changes in serum metabolic marker levels before and after orthognathic surgery. Values are presented as mean ± standard deviation (n = 28). Blood sample were taken preoperation (pre), 1 day (1 d), 1 week (1 w), 1 month (1 m), 3 months (3 m), and 6 months (6 m) after surgery. Values of all serum metabolic markers at each time versus preoperative levels were compared with the Tukey–Kramer HSD test for post hoc pair-wise comparisons (*p < 0.05). Abbreviations: CRP, C-reactive protein; ICTP, type I collagen C-telopeptide; ALP, alkaline phosphatase; BAP, bone-specific alkaline phosphatase.

Peripheral blood samples were taken preoperatively (Pre) and 1 day, 1 week, 1 month, 3 months, and 6 months postoperatively to analyse serum bone metabolic markers and circulating OCN^+^ cell percentages. Serum levels of C-reactive protein (CRP), an inflammatory marker, reached their maximum value 1 day (p < 0.05) after surgery and returned to the preoperative levels after 1 month. The maximum type I collagen C-telopeptide (ICTP) value, used as a bone resorption and osteoclastic marker, was observed 1 week (p < 0.05) after surgery and was significantly higher for 1 month (p < 0.05) before gradually decreasing to preoperative levels by 6 months. Alkaline phosphatase (ALP) and bone-specific alkaline phosphatase (BAP) levels, used as bone formation and osteoblastic activity markers, significantly decreased 1 day (p < 0.05) and from 1 day to 1 week (p < 0.05) after surgery, respectively, then increased to their maximum values by 1 month (p < 0.05) before decreasing gradually to preoperative levels by 6 months (Fig. 1).

### Changes in the proportion of OCN^+^ cells among peripheral blood mononuclear cells (PBMCs) after orthognathic surgery

**Figure 2:**
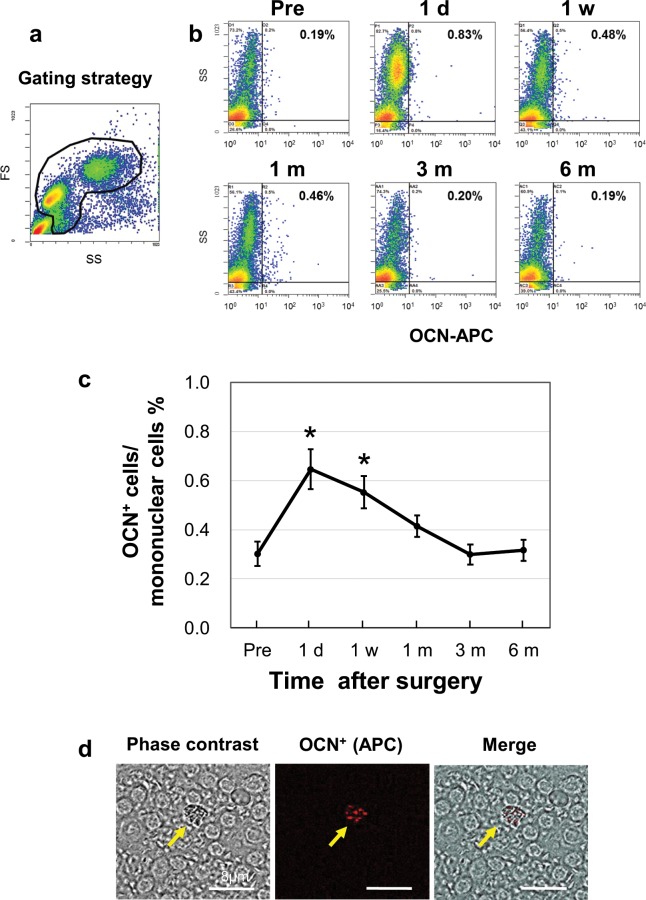
Proportion of osteocalcin positive (OCN^+^) cells among PBMCs. (**a**) Forward (FS)/side scatter (SS) profiles and gating strategy for analysis of flow cytometry data. The gate (black area) represents the mononuclear cells (the regions around the lymphocyte/monocyte-enriched area). (**b**) Proportion of circulating OCN^+^ cells among mononuclear cells of one patient before and after orthognathic surgery. The vertical axis shows SS and the horizontal axis shows the level of fluorescence intensity. (**c**) Changes in the percentage of OCN^+^ cells (p < 0.05) versus preoperative level. (**d**) Fluorescence microscopy of OCN^+^ cells.

The “lymphocyte-monocyte-enriched region” in forward/side scatters was gated for analysis (Fig. 2a) as previously described^9,15^. The proportion of circulating OCN^+^ cells among PBMCs of one representative patient before and after orthognathic surgery are shown (Fig. 2b). The proportion of OCN^+^ cells significantly increased from 1 day to 1 week after surgery (p < 0.05 versus preoperative level) and returned to the preoperative level 3 months after surgery (Fig. 2c). OCN^+^ cells were also confirmed by fluorescence microscopy (Fig. 2d).

### Osteocalcin is localised to both the plasma membrane and cytoplasm in OCN^+^ cells

**Figure 3:**
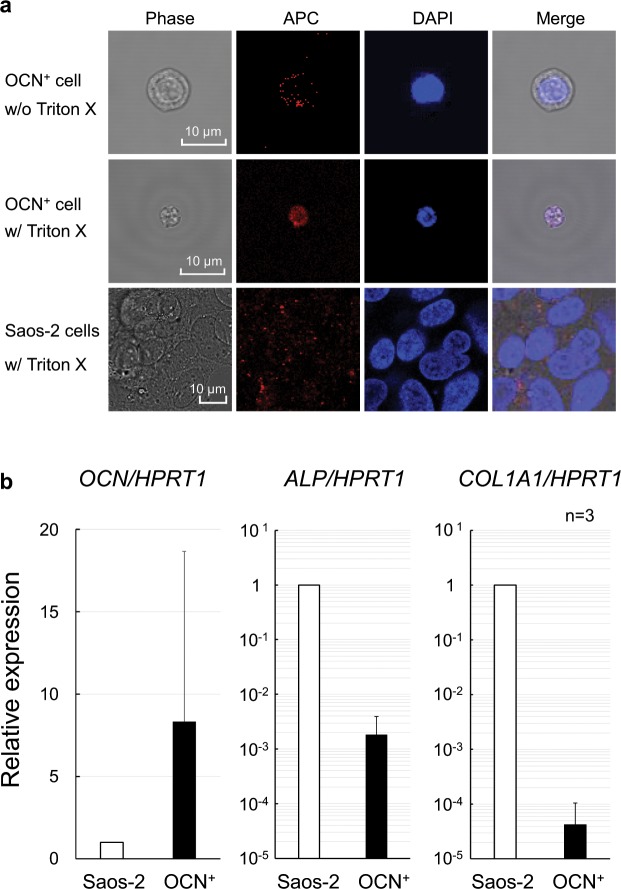
(**a**) Confocal microscopy of OCN^+^ cells. After fixation, sorted OCN^+^ cells by FACS were non-permeabilised (wo/Triton) or permeabilised with 0.2% Triton X-100 (w/Triton) and then stained with APC-conjugated anti-human osteocalcin (APC: red) antibody and DAPI (Nuclei: blue). Phase contrast image (Phase: grey) and merged images (Merge) are also shown. Saos-2 cells were used as a positive control of osteocalcin expression. Scale bar: 10 µm. (**b**) Real-time PCR quantification of bone-related gene expression of osteocalcin (*OCN*), bone alkaline phosphatase (*ALP*), and collagen type 1 alpha chain 1 (*COL1A1*). The graphs show the relative expression of FACS-sorted OCN^+^ cells normalised to Saos-2 cells. HPRT1 was used as the housekeeping gene. Values are presented as mean ± standard deviation (n = 3).

We examined OCN localisation using confocal microscopy. Compared with non-permeabilised cells, cells permeabilised with Triton-X100 were smaller and expressed OCN not only on their surfaces but also in the cytoplasm (Fig. 3a).

### Circulating OCN^+^ cells express bone-related genes

Finally, bone-related gene expression levels were analysed in OCN^+^ cells and compared with those of Saos-2 cells using real-time qPCR. The dissociation curve for both Saos-2 cells, as positive controls, and sorted OCN^+^ cells showed a single peak (Supplementary Fig. S1) representing a single amplification product. No signal was observed in case of the negative control, i.e., distilled H_2_O (Supplementary Fig. S1). These results demonstrated that the sorted OCN^+^ cells expressed *OCN*, *ALP*, and *COL1A1*. Figure 3b demonstrates the relative gene expression fold changes of *OCN*, *ALP*, and *COL1A1* in OCN^+^ cells after normalisation to the expression levels of Saos-2 cells (Fig. 3b).

## Discussion

Bone healing and bone metabolic change after orthognathic surgery (i.e. maxillary or/and mandibular osteotomy) are thought to be similar to those after bone fracture^16^. Studies of bone metabolic changes immediately after bone fracture have been reported^9,17,18^; however, since fractures occur accidentally, it is not possible to obtain baseline data for each bone metabolic parameter. Furthermore, it is unclear how much and how long the surgical intensity of orthognathic surgery actually causes bone metabolic change in humans. Thus, we clarified the time course of human bone metabolism and elucidated not only the catabolic but also the anabolic mechanism in the phases of the bone healing process after orthognathic surgery.

Firstly, we evaluated changes in serum markers over time after orthognathic surgery. CRP is a systemic inflammation marker that significantly increased relative to the preoperative level for 1 week, with the maximum value shown at 1 day after surgery before returning to preoperative levels after 1 month. Additionally, Miyaoka *et al*.^19^ reported that CRP, L-6, and IL-10 levels significantly increased 1 day after orthognathic surgery. Surgery-induced inflammation may activate monocytes, macrophages, and secretion of IL-1, IL-6, and tumour necrosis factor (TNF)-α from these cells. Circulating IL-6 produced in osteotomy sites binds hepatocytes, and CRP is produced by the liver^20^. These proinflammatory cytokines are also known as osteoclast-activating factors and may activate osteoclastogenesis and bone resorption throughout the body.

ICTP is a marker of bone resorption and a product of osteoclast-mediated degradation of type I collagen, a major organic component of bone^21^. ICTP significantly increased 1 week (1.97 fold) and 1 month (1.23 fold) after surgery suggesting active osteoclastic bone resorption and gradually decreased to preoperative levels by 6 months. By contrast, a previous report^22^ demonstrated a prolonged ICTP increase from 1 week (1.39 fold) to 3 months (1.15 fold) after surgery; however, all subjects analysed underwent two-jaw surgery. The extended phase of accelerated osteoclast activity in the previous report^22^ may have been caused by the degree of invasion, as all of their subjects underwent two-jaw surgery [Le Fort I osteotomy (LFI) of the maxilla and bilateral sagittal split osteotomy (BSSO) of the mandible], while our subjects included those who underwent either two-jaw or one-jaw (BSSO of the mandible) surgery. In future study, it will be necessary to investigate the effect of the degree of surgical invasion, such as two-jaw vs. one-jaw surgery.

Serum total ALP is a commonly used bone metabolism marker due to its simple application in subjects with normal liver function. ALP levels also indicate healthy new bone formation and osteoblast activity^23^. In addition, BAP isoenzyme (liver/bone/kidney, tissue non-specific, but modified specifically in bone) is a specific product of osteoblasts^24^.

Bowles *et al*.^17^ examined changes in total serum ALP and BAP for 14 days after bone fracture; total ALP continually increased, while BAP decreased and reached its nadir between days 4 and 8 before increasing. In this study, serum ALP and BAP significantly decreased 1 day after surgery; serum ALP began to increase from 1 week after surgery, whereas serum BAP significantly decreased 1 week after surgery. Subsequently, both ALP and BAP had significantly increased by 1 month and then gradually decreased to the preoperative level by 6 months. Notably, since normal preoperative levels of serum markers of patient before unexpected fracture are unknown, total ALP appears to rise compared to its value on the first day after fracture^17^. Thus, post-fracture and post- orthognathic surgery time courses show similar trends. This is the first report of the time course of serum ALP after surgical/fracture stress.

Consistent with our results, a reduction in serum BAP has been reported for 8–10 days followed by a subsequent increase for up to 1–2 months after both orthognathic surgery^25^ and fracture^17^. This decrease in serum BAP may be caused by surgical stress decreasing bone turnover or metabolic activity involving bone formation in early postoperative repair. Furthermore, ALP and BAP levels exhibited similar trends in our study. While BAP may be more sensitive, ALP may also indicate bone formation^23,24^.

Next, to integrally compare the dynamic changes in serum metabolic markers, OCN^+^ cells were studied. Cells expressing OCN^+^, ColI^+^, ALP^+^, and/or CD34^+^ are known to have osteogenic potential^18^. The percentage of OCN^+^ cells is significantly correlated with serum levels of bone turnover markers and bone formation both *in vitro* and *in vivo*; in contrast, the percentage of ALP^+^ cells was not correlated with these^9^. Meanwhile, CD34^+^ cells are capable of differentiating into osteoblastic cells *in vitro* and forming mineralised nodules, but CD34 expression is rapidly lost under osteoblast differentiating conditions *in vitro*, indicating that CD34^+^ cells represent a more primitive population^26,27^. For this reason, we focused on OCN as a marker for osteoblastic lineage cells. However, the roles of these markers in COP cells should be clarified in a future study.

The average percentage of OCN^+^ cells among PBMCs from preoperative patients (23.0 ± 5.4 y) was 0.31% in this study. Previous studies of the percentage of OCN^+^ cells reported 0.93% among PBMCs in patients aged 28–49 y^9^, 3.32% among PBMCs in healthy adults aged 26.3 ± 5.4 y^28^, and 0.42% (range 0.1–3.8%) of PBMCs in a healthy population aged 20–90 y (average age 55.5)^29^. This difference may be the result of variations in donor age^26,29^. Otherwise, it may be caused by the affinity of the antibody used for flow cytometry. We were not able to test the SC V-19 osteocalcin antibody, as it is commercially unavailable.

The proportion of OCN^+^ cells significantly increased from 1 day (2.16-fold vs. preoperative level) to 1 week after surgery and then returned to preoperative levels over 3 months in the present study. A previous study demonstrated that OCN^+^ cells significantly increased in adult patients with fracture^9^. By contrast, there were no significant variations in the number of circulating osteoblast precursors (CD15^−^ALP^+^OCN^+^) between baseline and 15 days after fracture^30^. Notably, no report has yet examined OCN^+^ cells at over 1 month after bone injury, such as fracture or surgery, but our study demonstrated changes in levels of circulating OCN^+^ cells before and up to 6 months after surgery.

Furthermore, we analysed the osteoblastic character of OCN^+^ cells by confocal microscopy and real-time PCR using Saos-2 cells as a positive control, because Saos-2 cells, which are human osteosarcoma cells, exhibit a mature osteoblastic phenotype in humans; are positive for OCN, ALP, and type I collagen; and are commonly used as an osteoblastic model^31^. Finally, we revealed that circulating OCN^+^ cells after orthognathic surgery express OCN protein not only on the cell membrane surface but also in the cytoplasm, suggesting that OCN may be synthesised by OCN^+^ cells themselves (Fig. 3a). Furthermore, these cells expressed bone-related genes (osteoblast markers), such as *OCN, ALP*, and *COL1A1* (Fig. 3b and Supplementary Fig. S1), demonstrating similar characteristics to those of COP cells^9,18^. However, OCN^+^ cells in PBMCs expressed a high level of *OCN*, low levels of *ALP* and type I collagen (*COL1A1*) mRNA compared with Saos-2 cells in this study. Circulating OCN^+^ cells increased after orthognathic surgery and may be recruited to the osteotomy site to participate in bone healing osteogenesis.

Our results of gene expression analysis in comparison with that in Saos-2 cells suggested that the increased circulating OCN^+^ cells after orthognathic surgery may not be able to form bone by themselves, as they do not have enough ALP, which increases the phosphate concentration in the process of calcification^32^, nor enough type I collagen, which is the main component of the bone extracellular matrix^33^. However, once these cells home in on the site of bone repair, they may differentiate into bone-forming osteoblasts and express ALP and type I collagen according to their differentiation stage^32,34,35^. Thus, as previously described in an *in vivo* transplant assay^9^, circulating OCN^+^ cells may form bone.

**Figure 4:**
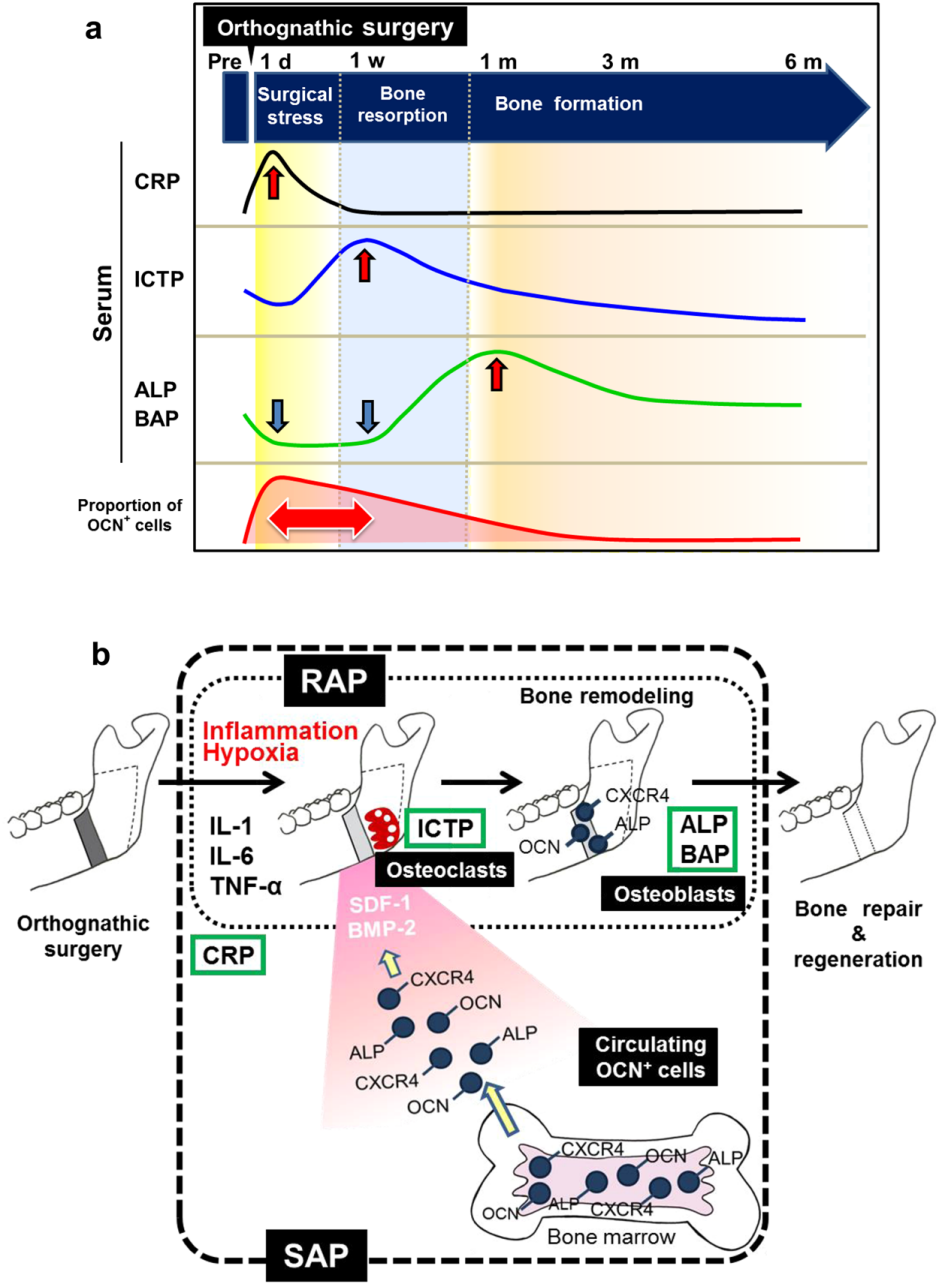
(**a**) Results summary. (**b**) Putative mechanism of COP (circulating osteogenic precursor) cell homing during bone repair after orthognathic surgery.

As a clinical implication, we would like to propose a model (Fig. 4) in which the acceleratory phenomena of bone metabolic activity after orthognathic surgery may induce rapid tooth movement. When surgical inflammation-induced RAP activates monocytes and macrophages, they secrete proinflammatory cytokines, including IL-1, TNF-α, and IL-6, that induce CRP production in the liver. These cytokines, called osteoclast-activating factors, induce osteoclast differentiation, maturation, activation, and survival^20^. Orthodontic tooth movement occurs due to bone resorption by osteoclasts on the compression side and bone formation by osteoblasts on the tension side. These circulating osteoclast-activating factors are thought to further induce osteoclasts to promote bone resorption on the compression side, promoting orthodontic tooth movement. At the same time, osteoblast precursor cells expressing OCN on their surfaces are supplied via circulation to the tension side to promote bone formation, presumably accelerating orthodontic tooth movement further.

It has been reported that the surgery-first approach to orthognathic surgery eliminates the presurgical orthodontics phase and shortens the treatment period compared to that of the conventional method^22,36,37^ Liou *et al*.^22^ firstly proposed the clinical phenomenon of postoperative rapid orthodontic tooth movement, explaining the increased tooth mobility and higher osteoclastic activity for 3–4 months postoperatively. In a preclinical histomorphometric study, RAP after surgery, such as alveolar corticotomy or orthognathic surgery, has been shown to increase the rate of orthodontic tooth movement in dogs^38^ or rodents^39^, respectively. At present, although there is no evidence regarding the increasing rate of orthodontic tooth movement after orthognathic surgery in humans, our study supports the hypothesis that the acceleration of orthodontic tooth movement and reduction of treatment period in the surgery-first approach are influenced not only by advantages in decompensation^40^ reducing soft tissue pressure after changing the jaw position, but also by postoperative RAP and SAP. Benefits of orthodontic tooth movement may reduce the total treatment time of maxillofacial deformities. Even so, it should be noted that the active phase of bone metabolism after orthognathic surgery is prolonged by at least more than 1 month and then gradually decreases at 3 months with no significant differences until it reaches almost the preoperative level at 6 months.

In conclusion, orthognathic surgery induces systemic dynamic changes in bone metabolic activity, particularly by targeting steps in the bone healing process, such as increasing CRP levels (inflammation marker) following surgical stress. This leads to a rise in ICTP (associated with osteoclastic activity), indicating bone resorption, followed by bone formation along with increases in ALP and BAP levels (associated with osteoblastic activity). During the early post-operative period, OCN^+^ cells, as COP cells in peripheral blood, may be involved in the SAP and may induce local OCN^+^ cell maturation and bone formation (Fig. 4a). This suggests that OCN^+^ cells are involved in the bone healing process after orthognathic surgery.

Finally, we propose a putative mechanism of bone healing after orthognathic surgery based on our findings and previous reports^1–3,9–14,18–20,26^. Once orthognathic surgery is performed, hypoxia and inflammation occur and induce proinflammatory cytokines, including IL-1, IL-6 and TNF-α, and production of CRP. Next, ICTP, which indicates osteoclastic activity, increases. By contrast, induced SDF-1 and BMPs form a chemoattractant gradient and recruit COP cells, such as OCN^+^ cells (cells may also be CXCR4^+^ and ALP^+^) from bone marrow via the circulation and target them to osteotomy sites. Meanwhile, the OCN^+^ cells inducing the SAP are released from the bone marrow into the circulation and to the osteotomy site. This process accelerates osteoblastic bone formation, which is followed by an increase in serum ALP and BAP levels. Finally, bone repair and bone regeneration are completed (Fig. 4b)

This study was conducted in humans and not in an animal model, and it provides important information regarding postoperative bone metabolism. Therefore, the results of this study will provide meaningful evidence to clinicians.

## Methods

### Ethics statement

This longitudinal, prospective observational study was approved by the Ethics Committee of Tohoku University Graduate School of Dentistry (2015-3-011) and all methods were performed in accordance with relevant guidelines and regulations. All patients and/or the guardians of patients younger than 20 years old provided written, informed consent.

### Patients and sample collection

The study included 28 consecutive patients (10 men and 18 women, 18–40 years of age, mean age: 23.0 ± 5.4 years) undergoing orthognathic surgery at the Division of Oral and Maxillofacial Surgery of Tohoku University Hospital (Sendai, Japan) between July 2016 and August 2017. No patient showed signs of infection or metabolic abnormality. Sixteen underwent LFI of the maxilla and BSSO of the mandible; 1 underwent LFI, BSSO, and genioplasty; and 11 underwent BSSO.

Peripheral blood samples were taken preoperatively (Pre) and 1 day, 1 week, 1 month, 3 months, and 6 months postoperatively to analyse serum bone metabolic markers and circulating OCN^+^ cell percentage.

### Serum bone metabolic markers

Serum levels of CRP, ICTP, ALP, and BAP were measured by N-assay LA CRP-S Nittobo D-Type (Nittobo Medical Co., Ltd., Tokyo, Japan) using a latex agglomeration method, Pirijinorin ICTP (Fujirebio, Inc., Gent, Belgium) using an RIA2 antibody assay, L-type Wako ALP J2 (Wako Pure Chemical Industries, Ltd., Osaka, Japan) with a colorimetric method, and Access Ostase (Beckman Coulter, Brea, CA, USA) with a chemiluminescent enzyme immunoassay (CLEIA), respectively. All serum assay procedures were performed by BML, Inc. (Tokyo, Japan).

### Flow cytometry, fluorescent microscopy, and fluorescence-activated cell sorter analyses of OCN^+^ cells

Samples from 18 of 28 patients operated on after December 2016 (12 underwent 2-jaw surgery, and 6 underwent 1-jaw surgery) were examined for OCN^+^ cells. Heparinised whole-blood samples were diluted 1:2 in PBS, layered over Ficoll-Paque PLUS (GE Healthcare, Little Chalfont, UK) density gradients, and centrifuged. PBMCs were collected and washed twice with PBS, and then processed for immunostaining and detection of OCN^+^ cells by flow cytometry. After having their nonspecific binding sites blocked with 2% bovine serum albumin (BSA) at room temperature (RT; 20 ± 5 °C) for 30 min, the PBMCs were incubated with mouse anti-human osteocalcin-APC (R&D Systems, Minneapolis, MN, USA) or mouse control IgG1-APC (R&D Systems) as an isotype control in the dark at 4 °C for 60 min, and then washed with PBS (4 °C, 400 *g* for 5 min). The cells were analysed using a flow cytometer (Cytomics FC500; Beckman Coulter) and software (CXP; Beckman Coulter). Twenty thousand events were counted with the gate for each sample. We used forward/side scatter to set regions around the lymphocyte/monocyte-enriched (Fig. 2b) and granulocyte-enriched areas. The gates were set to select and analyse the lymphocyte-monocyte-enriched region and identify the positive populations as cells that expressed specific levels of fluorescence activity above the nonspecific autofluorescence of the isotype control, as previously described^9,15^. The frequency of positive cells was measured as the percentage of gated cells in fluorescent channels with activities above 99.5% of the corresponding isotype controls, including backgrounds below 0.5%. APC-labelled OCN^+^ cells were visualised with a fluorescence microscope (BZ-9000; Keyence, Osaka, Japan). OCN^+^ cells were sorted using a FACS Aria II flow cytometer (BD Biosciences, Franklin Lakes, NJ, USA) and BD FACS Diva v6.1.3 (BD Biosciences).

### Saos-2 osteosarcoma cells

For the positive control of osteocalcin protein and gene expression, the human osteosarcoma-derived osteoblast cell line Saos-2, which exhibits most features of a mature osteoblastic profile^31^, was obtained from The Cell Resource Center for Biomedical Research (IDAC, Tohoku University, Miyagi, Japan). Cells were cultured in α-modified minimal essential medium (*α*-MEM; Wako) supplemented with 10% (v/v) foetal bovine serum (BioWest, Nuaillé, France), 100 U/mL of penicillin, and 100 μg/mL of streptomycin (Invitrogen, Carlsbad, CA, USA). The cells were incubated at 37 °C in a 5% CO_2_ environment. Confluent cells were passaged with trypsin-EDTA (Wako) and seeded at 6 × 10^6^ cells per 35-mm plastic culture dish (Falcon; Thermo Fisher Scientific, Waltham, MA, USA). Cells cultured for 1 day were used for confocal microscopy observation, and cells cultured for 3 days were used for total RNA extraction and subsequent real-time PCR analysis.

### Confocal microscopy

Sorted OCN^+^ cells were fixed overnight by 4% paraformaldehyde in PBS. On the following day, they were washed with PBS, permeabilised with 0.2% Triton X-100 (Sigma-Aldrich, St. Louis, MO, USA) for 15 min at RT, and then washed again with PBS. The cells were stained with mouse anti-human osteocalcin APC-conjugated antibody, the same antibody used for flow cytometry, then washed with PBS twice and stained with DAPI (Dojindo Molecular Technologies, Inc., Rockville, MD, USA) for nuclear staining before washing with PBS. Images were taken using a confocal microscope (TCS SP8; Leica Microsystems, Wetzlar, Germany) DMI6000 LASX 1.8 (Leica).

### RNA extraction and real-time reverse-transcription polymerase chain reaction (PCR)

Total RNA was extracted from sorted OCN^+^ cells using the RNeasy Micro kit (QIAGEN, Hilden, Germany). RNA purity and quantity were measured with a NanoDrop spectrophotometer (Thermo Fisher Scientific). Extracted total RNA from one or two patients was subjected to complementary DNA synthesis by reverse transcription with a PrimeScript RT reagent Kit (Takara Bio, Inc., Kusatsu, Japan) according to the manufacturer’s instructions. Real-time PCR analyses were performed using a Takara Thermal Cycler Dice Realtime System apparatus with SYBR Premix Ex Taq II (Takara). Specific primers (Supplementary Table S1) were employed for human *OCN*, *ALP*, and collagen type I alpha chain 1 (*COL1A1*) as bone-related genes, and *HPRT1* as the housekeeping gene, according to the manufacturer’s instructions. Following amplification, amplicon specificity and purity were confirmed by dissociation curve analysis. The relative expression of each gene was normalised to the expression levels of Saos-2 cells.

### Statistical analysis

All data are represented as mean ± standard deviation. Data were statistically analysed by repeated measures ANOVA followed by Tukey-Kramer HSD using JMP version 13.1 (SAS Institute, Inc., Cary, NC, USA). The level of significance was set at 0.05.

## Supplementary information

Dateset 1

## Data Availability

The datasets generated during and/or analysed during the current study are available from the corresponding author on reasonable request.
