# Supplementary material for: Increase in bone metabolic markers and circulating osteoblast-lineage cells after orthognathic surgery
Source: Sci Rep. 2019 Dec 27;9:20106. doi: 10.1038/s41598-019-56484-x (PMC6934478; doi:10.1038/s41598-019-56484-x)
Supplement: Supplementary file 1 — Dateset 1 [file 41598_2019_56484_MOESM1_ESM.pdf]

Supplymentaly Date

Increase in bone metabolic markers and circulating osteoblast-lineage cells after orthognathic surgery

Yoko Abe[1,2], Mirei Chiba[1*], Sanicha Yaklai[1,2], Roan Solis Pechayco[1,2], Hikari Suzuki[2], Tetsu Takahashi[2]

[1] Division of Oral Physiology, Department of Oral Function and Morphology, Graduate School of Dentistry, Tohoku University, 4-1 Seiryo-machi, Aoba-ku, Sendai 980-8575, Japan

[2] Division of Oral and Maxillofacial Surgery, Department of Oral Medicine and Surgery, Graduate School of Dentistry, Tohoku University, 4-1 Seiryo-machi, Aoba-ku, Sendai 980-8575, Japan

*Corresponding author: Mirei Chiba, DDS, PhD (E-mail: mirei.chiba.d6@tohoku.ac.jp)

The PDF files includes:

Supplementary Figure S1

Supplementary Table S1

Supplementary Figure S1

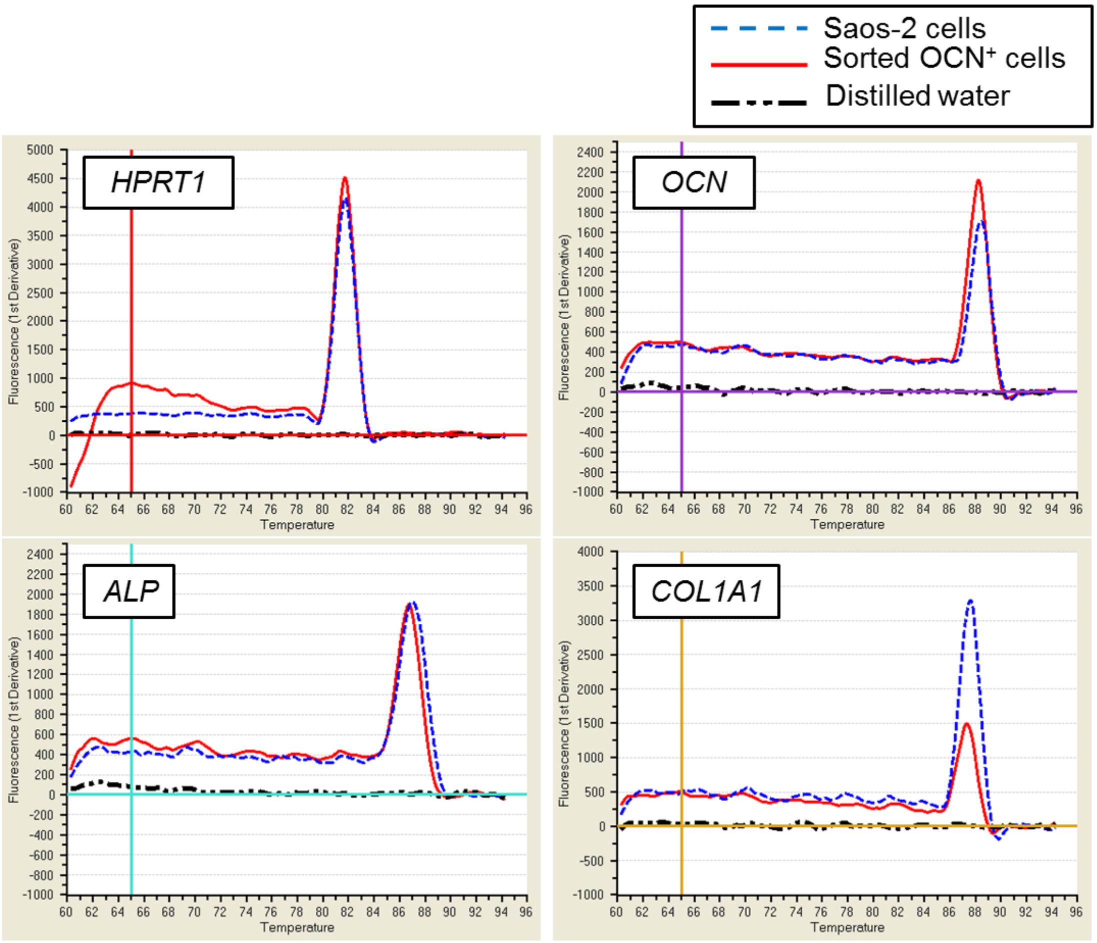

Supplementary Figure S1. Real-time PCR.

Dissociation curve analysis of Saos-2 cells as positive controls and FACS-sorted OCN$^+$ cells (*HPRT* was used as a housekeeping gene; *OCN*: osteocalcin, *ALP*: alkaline phosphatase, *COL1A1*: collagen type I alpha chain 1). The dissociation curves of Saos-2 cells (positive control)has been indicated by a blue dashed line, sorted OCN$^+$ cells by a red line, and distilled water (negative control) by a black dashed/dotted line. These dissociation curves were representative data of three independent experiments.

Supplementary Table S1

Gene	GenBank Accession No.	Primer Sequence	
		Forward (5' -> 3')	Reverse (5' -> 3')
HPRT1	NM 000194.2	GGCAGTATAATCCAAAGATGGTCAA	GTCAAGGGCATATCCTACAACAAAC
OCN	NM_199173	CACTCCTCGCCCTATTGGC	CCCTCCTGCTTGGACACAAAG
ALP	NM_001127501	ACCACCACGAGAGTGAACCA	CGTTGTCTGAGTACCAGTCCC
COL1A1	NM_000088	GAGGGCCAAGACGAAGACATC	CAGATCACGTCATCGCACAAC

Supplementary Table S1. Primer sequences used in this study.

Abbreviations: *HPRT1*, hypoxanthine phosphoribosyltransferase 1; *OCN*, osteocalcin; *ALP*, alkaline phosphatase; *COL1A1*, collagen type I alpha chain 1
